# Inappropriate direct oral anticoagulant dosing in atrial fibrillation patients is associated with prescriptions for outpatients rather than inpatients: a single-center retrospective cohort study

**DOI:** 10.1186/s40780-020-0157-z

**Published:** 2020-02-11

**Authors:** Motoyasu Miyazaki, Koichi Matsuo, Masanobu Uchiyama, Yoshihiko Nakamura, Yuya Sakamoto, Momoko Misaki, Kaoko Tokura, Shiro Jimi, Keisuke Okamura, Sen Adachi, Tomohiko Yamamoto, Kazuyuki Shirai, Hidenori Urata, Osamu Imakyure

**Affiliations:** 1 Department of Pharmacy, Fukuoka University Chikushi Hospital, Fukuoka Japan; 1-1-1, Zokumyoin, Chikushino-shi, Fukuoka, 818-8502 Japan; 2 Department of Emergency and Critical Care Medicine, Faculty of Medicine, Fukuoka University, Fukuoka, Japan; 7-45-1, Nanakuma, Jonan-ku, Fukuoka, 814-0180 Japan; 3 Central Laboratory for Pathology and Morphology, Faculty of Medicine, Fukuoka University, Fukuoka, Japan; 7-45-1, Nanakuma, Jonan-ku, Fukuoka, 814-0180 Japan; 4 Department of Cardiovascular Diseases, Fukuoka University Chikushi Hospital, Fukuoka, Japan; 1-1-1, Zokumyoin, Chikushino-shi, Fukuoka, 818-8502 Japan

**Keywords:** Direct oral anticoagulant, Inappropriate dose, Prescription, Outpatient

## Abstract

**Background:**

Inappropriate dosing of direct oral anticoagulants (DOACs) has been associated with clinical safety and efficacy; however, little is known about clinical data associated with an inappropriate DOAC dosing in Japan. In addition, there is no report in which the appropriateness of DOAC dosing between prescription for inpatients and for outpatients was examined. In this study, we aimed to investigate the prevalence and factors associated in the inappropriate dosing of DOACs in patients with atrial fibrillation (AF).

**Methods:**

The retrospective cohort study was conducted at a single Japanese university hospital. Both inpatients and outpatients, who were diagnosed with AF and for whom treatment with either dabigatran, rivaroxaban, apixaban, or edoxaban was initiated between April 1, 2014 and March 31, 2018, were enrolled in the study. Appropriateness of DOAC dosing was assessed according to the manufacturer’s labeling recommendations (dose reduction criteria) of each DOAC. Inappropriate reduced dose, namely, underdosing, was defined as prescription of a reduced dose of DOAC despite the patient not meeting the dose reduction criteria. Inappropriate standard dose, namely, overdosing, was defined as prescription of a standard dose of DOAC despite the patient meeting the dose reduction criteria. Inappropriate DOAC dosing was defined as a deviation of the recommended dose (both underdosing and overdosing).

**Results:**

A total of 316 patients (dabigatran, 28; rivaroxaban, 107; apixaban, 116; and edoxaban, 65) were included, with a median (interquartile range) age of 75 (66–81) years and 62.3% male. DOACs were prescribed at an appropriate standard dose in 39.2% of patients, an appropriate reduced dose in 36.7%, an inappropriate standard dose in 2.5%, and an inappropriate reduced dose in 19.3%. Multivariate analysis revealed that the inappropriate dosing of DOACs was significantly associated with prescriptions for outpatients (vs. inpatients; odds ratio [OR] 2.87, 95% confidence interval [CI] 1.53–5.62, *p* < 0.001) and those with higher HAS-BLED scores (OR 1.87, 95% CI 1.42–2.51, p < 0.001).

**Conclusions:**

Our results demonstrated that the inappropriate dosing of DOACs occurred in approximately 20% of AF patients, and was more frequent in outpatients (vs. inpatients) and in those with a higher risk of bleeding. It is recommended that pharmacists play a greater role in assisting in the prescription process to help physicians make better decisions.

## Background

Atrial fibrillation (AF) is the most common serious arrhythmia and an important risk factor of cardiogenic stroke. In Japan, the prevalence of AF is predicted to increase to ≥1% of the Japanese population by 2050 [1]. Until recently, warfarin was the only agent used to prevent stroke in AF patients. However, since 2011, the direct oral anticoagulants (DOACs) dabigatran, rivaroxaban, apixaban, and edoxaban have been approved for anticoagulant therapy in Japan. A meta-analysis of randomized clinical trials has shown the benefit of DOAC therapy in reducing the incidence of stroke, intracranial hemorrhage, and mortality among AF patients, compared with the use of warfarin [2]. In addition, anticoagulant regimens involving warfarin are generally under-prescribed by physicians because of the fear of increased bleeding [3, 4]. In contrast, DOACs have fixed dose regimens and do not require frequent dose adjustments or routine pharmacodynamic monitoring, including the prothrombin time-international normalized ratio (PT-INR). However, the dosing of these drugs should be appropriately adjusted based on one or more clinical features such as renal function, age, body weight, and concomitant drug use. In Japan, the prescription dose for each DOAC is determined by the manufacturer’s recommendations in the drug package insert, and the physician’s prescription is usually audited by pharmacists; however, physicians occasionally prescribe inappropriate doses of DOACs in real-world clinical settings [5–7]. Because few hospitals in Japan describe clinical laboratory data for out-of-hospital prescriptions, proper dosing adjustments may not be performed with prescriptions for outpatients compared with those for inpatients [8]. However, to date, little is known in Japan about clinical factors associated with an inappropriate DOAC dosing, including whether it is prescribed for inpatients or outpatients. The inappropriate dosing of DOACs has been associated with clinical safety and efficacy [6, 9]. Therefore, it is essential to clarify the factors associated with the inappropriate dosing of DOACs to better educate physicians and improve their correct use.

The aim of the present study was to examine the prevalence and associated factors related to the inappropriate dosing of DOACs in AF patients at a single Japanese center.

## Methods

### Setting and study population

This retrospective cohort pilot study was conducted at a single Japanese university hospital. We included inpatients and outpatients attending the Department of Cardiovascular Diseases who were diagnosed with AF and for whom treatment with either dabigatran, rivaroxaban, apixaban, or edoxaban was initiated between April 1, 2014 and March 31, 2018 (from fiscal year 2014 to 2017). Patients who were under 20 years old, for whom a DOAC was initiated for the treatment or prophylaxis of venous thromboembolism, or who had history of DOAC prescription prior to the study period, were excluded.

### Data collection

We collected patient data when a DOAC was prescribed for the first time during the study period. Patient characteristics, clinical information, and prescription information after pharmacist’s inquiries (if any) were obtained from electronic medical records. These characteristics included age, gender, height, body weight, body mass index, current smoking, and alcohol abuse. Clinical information included comorbidities (hypertension, heart failure, myocardial infarction, dyslipidemia, diabetes mellitus, cerebrovascular disease, and hepatitis) and baseline laboratory data. Creatinine clearance (CrCl) was estimated using the Cockcroft and Gault formula [10]. History of bleeding, including gastrointestinal hemorrhage (GIH), was also obtained from the medical records. Patient prescription information included the following information: dosage and administration of DOAC; number of medicines; polypharmacy (number of oral medications ≥6) [11]; concomitant drug use, particularly antiplatelet agents, P-glycoprotein inhibitors, and hepatic cytochrome P450 3A4 inhibitors; and a history of warfarin use. CHADS_2_, CHA_2_DS_2_-VASc, and HAS-BLED scores were calculated for each patient based on his or her clinical data at the time of initial DOAC prescription [12–14].

### DOAC dosing

The appropriateness of DOAC dosing was assessed according to the recommendations of the drug package insert for each DOAC. We used certain dose reduction criteria, including renal function, age, body weight, concomitant drug use, and history of GIH, as listed in Table 1. An inappropriate reduced dose, namely, underdosing, was defined as a prescription for a reduced dose of a DOAC despite the patient not meeting the dose reduction criteria. An inappropriate standard dose, namely, overdosing, was defined as a prescription for a standard dose of a DOAC despite the patient meeting the dose reduction criteria. An inappropriate DOAC dosing was defined as a deviation of the recommended dose (i.e., both underdosing and overdosing). An off-label dose was defined as a dose not described in the drug insert package.

**Table 1:** Dose reduction criteria of dabigatran, rivaroxaban, apixaban, and edoxaban in our study

		Dabigatran	Rivaroxaban	Apixaban	Edoxaban
Standard dose		150 mg twice daily	15 mg once daily	5 mg twice daily	60 mg once daily
Reduced dose		110 mg twice daily	10 mg once daily	2.5 mg twice daily	30 mg once daily
Dose reduction criteria	Renal function	CrCl 30 to 50 mL/min	CrCl 15 to 49 mL/min	≥2 of the following: Serum creatinine ≥1.5 mg/dL	CrCl 15 to 50 mL/min
	Age	Age ≥ 70 years		Age ≥ 80 years	
	Body weight			Body weight ≤ 60 kg	Body weight ≤ 60 kg
Concomitant drug use		P-gp inhibitors (quinidine, verapamil, amiodarone, tacrolimus, cyclosporine, ritonavir, nelfinavir, saquinavir)	CYP 3A4 and P-gp inhibitors (fluconazole, fosfluconazole, clarithromycin, erythromycin)	CYP 3A4 and P-gp inhibitors [Azole antifungal agents (except for fluconazole)] and HIV protease inhibitors]	P-gp inhibitors (quinidine, verapamil, cyclosporine, erythromycin, azithromycin, clarithromycin, itraconazole, diltiazem, amiodarone, HIV protease inhibitors)
	Other	History of GIH			

*CrCl*, Creatinine clearance; *CYP*, hepatic cytochrome P450; *GIH*, Gastrointestinal hemorrhage; *HIV*, Human immunodeficiency virus; *P-gp*, P-glycoprotein

### Statistical analyses

Binary variables were expressed as proportions and continuous variables were expressed as the medians and interquartile ranges (IQRs). Differences in the continuous variables among the four DOACs were evaluated using the Steel–Dwass test, with differences in categorical variables evaluated using the chi–squared test. Significance was adjusted for multiple comparisons using Bonferroni correction. To determine the factors associated with the inappropriate dosing of DOACs, comparisons between appropriate and inappropriate DOAC dosing groups were conducted by univariate analysis using the chi–squared test or Fisher’s exact test (as appropriate) for proportions and the Mann–Whitney U test for medians. Factors that were significantly associated with the inappropriate dosing of DOACs in univariate analysis were included in multivariate logistic regression analysis via a stepwise procedure to identify risk factors that were independently associated with inappropriate dosing. A trend analysis for the appropriateness of DOAC dosing was performed using the Cochran–Armitage trend test. All statistical analyses were performed using JMP® 14 (SAS Institute Inc., Cary, NC, USA), with a *p* value < 0.05 considered significant.

## Results

### Baseline characteristics

A total of 316 patients (118 inpatients and 198 outpatients) were included in this study, with a median (IQR) age of 75 (66–81) years and 62.3% male. The demographic characteristics of the patients stratified by DOAC are listed in Table 2. Patients administered apixaban (median [IQR]: 79 [73–83] years) were older than those administered dabigatran (71 [65–80] years) and rivaroxaban (71 [65–79] years) (*p* = 0.434 and *p* < 0.001, respectively). In addition, median (IQR) CrCl was lower in patients administered apixaban (51.3 [40.1–64.7] mL/min) than in those administered dabigatran (64.4 [48.5–82.1] mL/min) and rivaroxaban (60.9 [50.2–82.5] mL/min) (*p* = 0.079 and *p* = 0.002, respectively). Overall, 200 (64.1%) of the 316 patients had a CrCl ≥50 mL/min. There were no differences in comorbidities among the patients given the four DOACs. Overall, approximately 70% of patients presented with hypertension, greater than 30% with heart failure and dyslipidemia, 25% with diabetes mellitus, and greater than 10% with myocardial infarction and cerebrovascular disease. Ninety-two (29.1%) of the 316 patients had a history of warfarin use and 79 (25%) had concomitant antiplatelet drugs. The proportion of patients who smoked was higher in the rivaroxaban group (18.7%) than in the apixaban group (6.9%) (*p* = 0.047). Median (IQR) CHADS_2_ and CHA_2_DS_2_-VASc scores were higher in patients administered apixaban (2 [1–3] and 4 [3–5], respectively) than in those administered rivaroxaban (1 [1–3] and 3 [2–4], respectively) (*p* = 0.006 and *p* = 0.010, respectively). There were no significant differences in the HAS-BLED scores among the patients given the four DOACs.

**Table 2:** Demographic characteristics of the patients stratified by direct oral anticoagulant

Characteristics	Overall (*n* = 316)	Dabigatran (*n* = 28)	Rivaroxaban (*n* = 107)	Apixaban (*n* = 116)	Edoxaban (*n* = 65)
Age in years, median (IQR)	75 (66–81)	71 (65–80)	71 (65–79)	79 (73–83)	75 (65–81)
< 65	57 (18.0)	6 (21.4)	24 (22.4)	13 (11.2)	14 (21.5)
65 ≤ − < 70	48 (15.2)	6 (21.4)	25 (23.4)	9 (7.8)	8 (12.3)
70 ≤ − < 75	46 (14.6)	3 (10.7)	16 (15.0)	18 (15.5)	9 (13.8)
75 ≤ − < 80	65 (20.6)	6 (21.4)	16 (15.0)	26 (22.4)	17 (26.2)
80≤	100 (31.6)	7 (25.0)	26 (24.3)	50 (43.1)	17 (26.2)
Male gender	197 (62.3)	22 (78.6)	72 (67.3)	67 (57.8)	36 (55.4)
Body weight in kg, median (IQR)	59.4 (50.6–68.0)^c^	61.6 (54.6–66.5)^a^	59.8 (51.9–70.0)^b^	57.7 (47.3–66.3)^a^	57.4 (50.4–66.9)
BMI in kg/m2, median (IQR)	23.0 (20.9–25.0)^d^	22.9 (21.7–25.6)^a^	23.7 (21.6–25.2)^b^	22.7 (20.3–24.6)^b^	22.4 (20.3–24.9)
< 18.5	24 (7.7)	1 (3.7)	3 (2.9)	13 (11.4)	7 (10.8)
18.5 ≤ − < 25	210 (67.5)	19 (70.4)	73 (69.5)	76 (66.7)	42 (64.6)
25 ≤ − < 30	67 (21.5)	7 (25.9)	26 (24.8)	19 (16.7)	15 (23.1)
30≤	10 (3.2)	0 (0)	3 (2.9)	6 (5.3)	1 (1.5)
Type of hospital visit					
Inpatients	118 (37.3)	5 (17.9)	32 (29.9)	51 (44.0)	30 (46.2)
Outpatients	198 (62.7)	23 (82.1)	75 (70.1)	65 (56.0)	35 (53.8)
CrCl in mL/min, median (IQR)	57.7 (43.9–78.5)^c^	64.4 (48.5–82.1)^a^	60.9 (50.2–82.5)^b^	51.3 (40.1–64.7)^a^	60.8 (43.3–94.4)
50≤	200 (64.1)	19 (70.4)	80 (76.2)	61 (53.0)	40 (61.5)
30 ≤ − < 50	90 (28.8)	8 (29.6)	21 (20.0)	44 (38.3)	17 (26.2)
15 ≤ − < 30	21 (6.7)	0 (0)	3 (2.9)	10 (8.7)	8 (12.3)
< 15	1 (0.3)	0 (0)	1 (1.0)	0 (0)	0 (0)
Alcohol abuse	11 (3.5)	2 (7.1)	4 (3.7)	3 (2.6)	2 (3.1)
Smoking	41 (13.0)	4 (14.3)	20 (18.7)	8 (6.9)	9 (13.8)
History of warfarin use	92 (29.1)	10 (35.7)	29 (27.1)	40 (34.5)	13 (20.0)
History of bleeding	42 (13.3)	4 (14.3)	13 (12.1)	19 (16.4)	6 (9.2)
History of GIH	14 (4.4)	3 (10.7)	2 (1.9)	8 (6.9)	1 (1.5)
Comorbidities					
Hypertension	210 (66.5)	19 (67.9)	68 (63.6)	75 (64.7)	48 (73.8)
Heart failure	99 (31.3)	7 (25.0)	28 (26.2)	41 (35.3)	23 (35.4)
Myocardial infarction	32 (10.1)	3 (10.7)	14 (13.1)	11 (9.5)	4 (6.2)
Dyslipidemia	104 (32.9)	13 (46.4)	37 (34.6)	29 (25.0)	25 (38.5)
Diabetes mellitus	79 (25.0)	8 (28.6)	24 (22.4)	31 (26.7)	16 (24.6)
Cerebrovascular disease	41 (13.0)	3 (10.7)	11 (10.3)	19 (16.4)	8 (12.3)
Hepatitis	17 (5.4)	0 (0)	7 (6.5)	5 (4.3)	5 (7.7)
Polypharmacy	129 (40.8)	9 (32.1)	37 (34.6)	57 (49.1)	26 (40.0)
Concomitant drug use					
Antiplatelet drug	79 (25.0)	3 (10.7)	36 (33.6)	25 (21.6)	15 (23.1)
SAPT	55 (17.4)	2 (7.1)	27 (25.2)	16 (13.8)	10 (15.4)
DAPT	13 (4.1)	1 (3.6)	4 (3.7)	4 (3.4)	4 (6.2)
Non-SAPT/DAPT	17 (5.4)	0 (0)	9 (8.4)	6 (5.2)	2 (3.1)
NSAIDs	4 (1.3)	0 (0)	1 (0.9)	2 (1.7)	1 (1.5)
Amiodarone	11 (3.5)	2 (7.1)	1 (0.9)	4 (3.4)	4 (6.2)
Verapamil	4 (1.3)	0 (0)	4 (3.7)	0 (0)	0 (0)
Diltiazem	11 (3.5)	1 (3.6)	2 (1.9)	7 (6.0)	1 (1.5)
CHADS_2_ score, median (IQR)	2 (1–3)	2 (1–3)	1 (1–3)	2 (1–3)	2 (1–3)
0–1	123 (38.9)	12 (42.9)	56 (52.3)	33 (28.4)	22 (33.8)
≥ 2	193 (61.1)	16 (57.1)	51 (47.7)	83 (71.6)	43 (66.2)
CHA_2_DS_2_-VASc score, median (IQR)	3 (2–5)	3 (2–4)	3 (2–4)	4 (3–5)	4 (2–5)
0–1	46 (14.6)	4 (14.3)	24 (22.4)	9 (7.8)	9 (13.8)
2–3	115 (36.4)	13 (46.4)	41 (38.3)	40 (34.5)	21 (32.3)
≥ 4	155 (49.1)	11 (39.3)	42 (39.3)	67 (57.8)	35 (53.8)
HAS-BLED score, median (IQR)	2 (1–2)	2 (1–2)	2 (1–2)	2 (1–2)	1 (1–2)
0–2	252 (79.7)	27 (96.4)	85 (79.4)	89 (76.7)	51 (78.5)
≥ 3	64 (20.3)	1 (3.6)	22 (20.6)	27 (23.3)	14 (21.5)

Missing data: ^a^ n-1, ^b^ n-2, ^c^ n-4, ^d^ n-5

Abbreviations: *BMI*, Body mass index; *CrCl*, Creatinine clearance; *DAPT*, Dual antiplatelet therapy; *GIH*, Gastrointestinal hemorrhage; *IQR*, Interquartile range; *NSAID*, Non-steroidal anti-inflammatory drug; *SAPT*, Single antiplatelet therapy

### Appropriateness of DOAC dosing

**Fig. 1:**
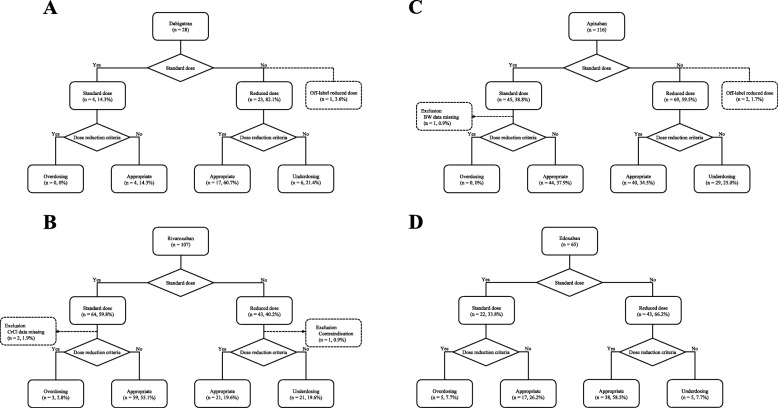
Appropriateness of dabigatran (**a**), rivaroxaban (**b**), apixaban (**c**), and edoxaban (**d**) dosing. BW: body weight: CrCl: creatinine clearance

**Fig. 2:**
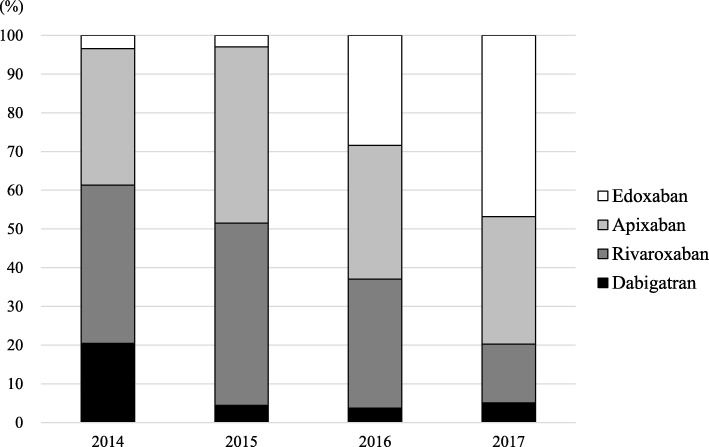
Trends in direct oral anticoagulant prescriptions from fiscal year 2014 to 2017

A total of 28 patients were prescribed dabigatran, 107 rivaroxaban, 116 apixaban, and 65 edoxaban (Fig. 1). The standard dose of the given DOAC was prescribed to 135 patients (42.7%), with a reduced dose given to 178 patients (56.3%). An off-label reduced dose was prescribed to one patient for dabigatran (75 mg twice daily) and two patients for apixaban (2.5 mg once daily). The use of a DOAC was contraindicated in only one patient for rivaroxaban, whose CrCl was 13.8 mL/min. The prescription rates of dabigatran and rivaroxaban have been significantly decreasing, with rates of 20.5 and 40.9% in 2014, 4.4 and 47.1% in 2015, 3.7 and 33.3% in 2016, and 5.1 and 15.2% in 2017, respectively (*p* < 0.001). In contrast the prescription rate of edoxaban has been significantly increasing, at 3.4% in 2014, 2.9% in 2015, 28.4% in 2016, and 46.8% in 2017 (p < 0.001) (Fig. 2).

**Fig. 3:**
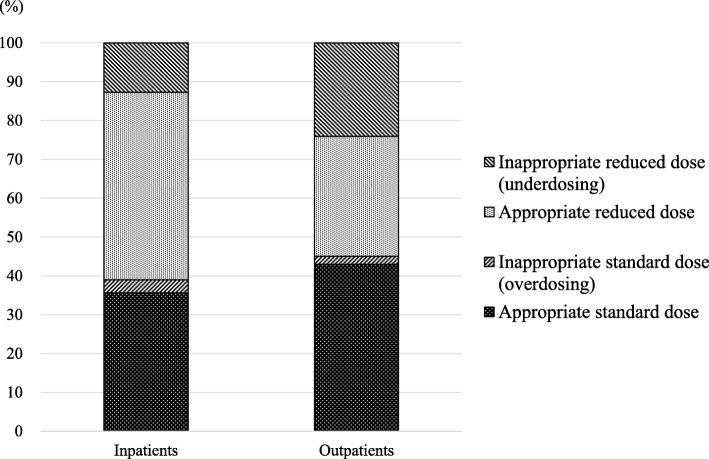
Rates of the appropriate standard dose, appropriate reduced dose, underdosing, and overdosing for both inpatients and outpatients

**Fig. 4:**
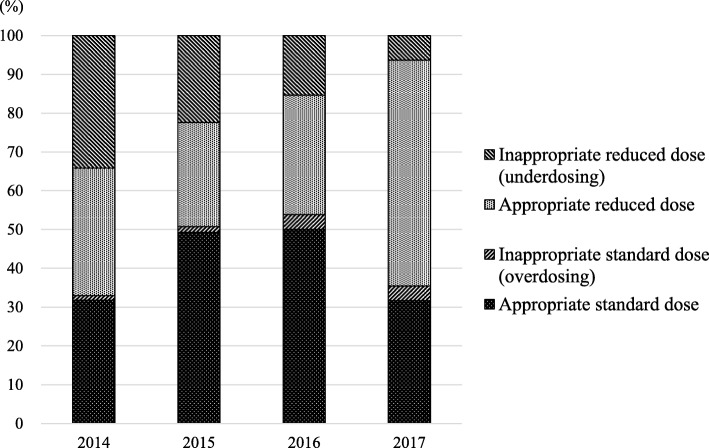
Trends in the appropriateness of direct oral anticoagulant dosing from fiscal year 2014 to 2017

Of the 316 patients included in this study, the appropriateness of DOAC dosing could be assessed by using dose reduction criteria in 309 (97.8%) patients. DOACs were prescribed at an appropriate standard dose in 124 (39.2%) patients and at an appropriate reduced dose in 116 (36.7%). Underdosing and overdosing occurred in 19.3 and 2.5% of patients, respectively. The appropriateness for each DOAC is described in Fig. 1. For all four DOACs, underdosing occurred more frequently than overdosing. In addition, the prevalence of underdosing tended to be higher for apixaban than for the other three DOACs (*p* = 0.066), while it was significantly lower for edoxaban than for the other three DOACs (*p* = 0.003). The rates of the appropriate standard dose, appropriate reduced dose, underdosing, and overdosing for both inpatients and outpatients are shown in Fig. 3. The rate of underdosing was significantly higher in outpatients than that in inpatients (24.1% vs. 12.7%, *p* = 0.015), and that of appropriate reduced dose was significantly lower in outpatients than that in inpatients (30.9% vs. 48.3%, *p* = 0.002). The appropriateness of DOAC dosing per fiscal year is shown in Fig. 4. The rate of underdosing appears to be significantly decreasing, with a rate of 34.1% in 2014, 22.4% in 2015, 15.4% in 2016, and 6.3% in 2017 (*p* < 0.001). During the study period, a total of 20 cardiologists initiated DOAC therapy for at least one or more patients, and we evaluated the appropriateness of DOAC dosing for 12 physicians who initiated DOAC therapy for more than 10 patients. The rate of underdosing varied among physicians, ranging from 0 to 45.5%, while overdosing ranged from 0 to 9.5% (Additional file 2: Figure S1).

### Determinants for inappropriate dosing of DOACs

The clinical features associated with the appropriateness of DOAC dosing are listed in Table 3. Inappropriate doses of DOAC were prescribed at a significant higher rate in outpatients compared with inpatients (72.5% vs. 58.8%, *p* = 0.039). Patients prescribed an inappropriate dose of a DOAC had higher rates of myocardial infarction (17.4% vs. 7.9%, *p* = 0.021) and cerebrovascular disease (20.3% vs. 10.8%, p = 0.039) as comorbidities, along with higher median (IQR) CHA_2_DS_2_-VASc (4 [3–5] vs. 3 [2–4], p = 0.021) and HAS-BLED (2 [1–3] vs. 1 [1, 2], *p* < 0.001) scores than those of patients administered an appropriate dose. Patients prescribed an inappropriate dose of DOAC tended to have a greater history of bleeding (20.3% vs. 11.7%, *p* = 0.066), polypharmacy (50.7% vs. 37.9%, *p* = 0.056), and higher median (IQR) CHADS_2_ score (2 [1–3] vs. 2 [1–3], *p* = 0.052). In addition, these patients tended to receive single antiplatelet therapy (24.6% vs. 15.4%, *p* = 0.076) at a higher rate than those given an appropriate dose. The comparison of demographic characteristics among the four groups (i.e., underdosing, appropriate reduced dosing, overdosing, and appropriate standard dosing of DOACs) are listed in Additional file 1: Table S1.

**Table 3:** Comparison of demographic characteristics between the appropriate and inappropriate dosing of direct oral anticoagulants

Characteristics	Appropriate dosing (*n* = 240)	Inappropriate dosing (*n* = 69)	P value*
Age in year, median (IQR)	75 (66–82)	75 (67–79)	0.710
Male gender	146 (60.8)	46 (66.7)	0.379
Body weight in kg, median (IQR)	57.7 (50.0–69.0)^a^	61.3 (52.2–65.5)	0.581
BMI in kg/m^2^, median (IQR)	22.9 (20.8–24.9)^a^	23.2 (20.9–24.9)^a^	0.616
Type of hospital visit			0.039
Inpatients	99 (41.3)	19 (27.5)	
Outpatients	141 (58.8)	50 (72.5)	
CrCl in mL/min, median (IQR)	58.4 (42.4–81.0)^a^	56.4 (48.9–71.4)	0.882
Alcohol abuse	7 (2.9)	3 (4.3)	0.699**
Smoking	32 (13.3)	8 (11.6)	0.705
History of warfarin use	66 (27.5)	24 (34.8)	0.241
History of bleeding	28 (11.7)	14 (20.3)	0.066
History of GIH	9 (3.8)	5 (7.2)	0.320**
DOAC			0.464
Dabigatran	21 (8.8)	6 (8.7)	
Rivaroxaban	80 (33.3)	24 (34.8)	
Apixaban	84 (35.0)	29 (42.0)	
Edoxaban	55 (22.9)	10 (14.5)	
Comorbidities			
Hypertension	157 (65.4)	49 (71.0)	0.385
Heart failure	73 (30.4)	26 (37.7)	0.254
Myocardial infarction	19 (7.9)	12 (17.4)	0.021
Dyslipidemia	79 (32.9)	24 (34.8)	0.772
Diabetes mellitus	58 (24.2)	19 (27.5)	0.569
Cerebrovascular disease	26 (10.8)	14 (20.3)	0.039
Hepatitis	12 (5.0)	3 (4.3)	> 0.999**
Polypharmacy	91 (37.9)	35 (50.7)	0.056
Concomitant drug use			
Antiplatelet drug	55 (22.9)	22 (31.9)	0.1291
SAPT	37 (15.4)	17 (24.6)	0.076
DAPT	10 (4.2)	3 (4.3)	> 0.999**
Non-SAPT/DAPT	11 (4.6)	4 (5.8)	0.751*
NSAIDs	3 (1.3)	1 (1.4)	> 0.999**
Amiodarone	8 (3.3)	3 (4.3)	0.714**
Verapamil	2 (0.8)	1 (1.4)	0.533**
Diltiazem	10 (4.2)	1 (1.4)	0.466**
CHADS_2_, median (IQR)	2 (1–3)	2 (1–3)	0.052
0–1	98 (40.8)	21 (30.4)	0.118
≥ 2	142 (59.2)	48 (69.6)	
CHA_2_DS_2_-VASc, median (IQR)	3 (2–4)	4 (3–5)	0.021
0–1	40 (16.7)	3 (4.3)	0.0312
2–3	87 (36.3)	27 (39.1)	
≥ 4	113 (47.1)	39 (56.5)	
HAS-BLED, median (IQR)	1 (1–2)	2 (1–3)	< 0.001
0–2	198 (82.5)	49 (71.0)	0.036
≥ 3	42 (17.5)	20 (29.0)	

Missing data: ^a^ n-1

Abbreviations: *BMI*, Body mass index; *CrCl*, Creatinine clearance; *DAPT*, Dual antiplatelet therapy; *DOAC*, Direct oral anticoagulants; *GIH*, Gastrointestinal hemorrhage; *IQR*, Interquartile range; *NSAID*, Non-steroidal anti-inflammatory drug; *SAPT*, Single antiplatelet therapy

*Comparison between the appropriate and inappropriate dosing of direct oral anticoagulants by using the chi–squared test or Fisher’s exact test (as appropriate) for proportions and the Mann–Whitney U test for medians

**Fisher’s exact test

Using multivariate analysis, we determined that the inappropriate dosing of DOACs was significantly associated with prescriptions for outpatients (vs. for inpatients; odds ratio [OR] 2.87, 95% confidence interval [CI] 1.53–5.62, *p* < 0.001) and higher HAS-BLED score (OR 1.87, 95% CI 1.42–2.51, p < 0.001) (Table 4).

**Table 4:** Factors associated with the inappropriate dosing of direct oral anticoagulants in multivariate analysis

Predictor variables	Odds ratio	95% confidence interval	P value
Outpatients (vs. inpatients)	2.87	1.53–5.62	< 0.001
HAS-BLED score*	1.87	1.42–2.51	< 0.001

*Odds ratio per point increase

Type of hospital visit (outpatients or inpatients), history of bleeding, myocardial infarction, cerebrovascular disease, polypharmacy, single antiplatelet therapy, CHADS_2_ score, CHA_2_DS_2_-VASc score, and HAS-BLED score, which were factors associated (*p* < 0.1) with inappropriate direct oral anticoagulant prescription in univariate analysis, were included in multivariate logistic regression analysis with a stepwise procedure

## Discussion

Although DOACs have made a significant contribution to anticoagulant therapy, the use of these drugs should still be carefully managed. In this study, we investigated the prevalence and factors associated with the inappropriate dosing of DOACs by cardiologists in a Japanese university hospital. The main findings of our study were that an inappropriate dose of DOACs was prescribed in 21.8% of patients, with underdosing (19.3%) being more common than overdosing (2.5%), and prescriptions for outpatients (vs. inpatients) and those with higher HAS-BLED scores were associated with inappropriate doses.

There are two large registries that show the prevalence of the inappropriate dosing of DOACs in Japan, namely, the Fushimi AF Registry [5] and the SAKURA AF Registry [6, 15]. In the Fushimi AF Registry, 32.2% (37/115) of dabigatran, 21.2% (47/222) of rivaroxaban, and 25.7% (52/202) of apixaban users were prescribed an off-label under-dose (i.e., underdosing) [5]. The SAKURA AF Registry revealed that inappropriate doses of DOACs were prescribed in 26.2% patients (underdosing in 22.2% and overdosing in 4.0% of patients, respectively) [6, 15]. Furthermore, a retrospective cohort study conducted at a single Japanese center showed that 22.6% of patients receiving DOACs were inappropriately prescribed, with 21.3% underdosed and 1.3% overdosed [7]. These results indicated that an inappropriate dose of a DOAC was prescribed in approximately 1 in 4 or 5 AF patients in Japan, which is in accordance with our results. In contrast, the ORBIT-AF II Registry, a nationwide AF registry conducted at a community practice in the US, demonstrated that an inappropriate dose of a DOAC was prescribed only in 12.5% (994/7925) of patients (underdosing in 9.3% [734/7925] and overdosing in 3.3% [260/7925] of patients, respectively) [9]. In a real-world registry in Spain, the rate of underdosing and overdosing of DOAC therapy was 17.5% (93/530) and 14.9% (79/530), respectively [16]. Other retrospective studies conducted abroad indicate that 5.4–17.4% patients are prescribed inappropriate reduced doses of DOACs (Additional file 1: Table S2) [17–20]. In our study, it was found that the rate of underdosing has been decreasing every year. This may be because the prescription rate of edoxaban has been increasing recently. For DOACs other than edoxaban, the appropriateness of dosing is evaluated by considering body weight as well as age and renal function. However, for edoxaban, doses may be determined only by body weight (≤60 kg); for example, in the case of a male patient, aged 65 years, with serum creatinine level of 0.8 mg/dL and body weight of 55 kg (CrCl of 72 mL/min), edoxaban meets the dose reduction criteria, whereas but the other DOACs do not. Therefore, the rate of appropriate reduced doses may be higher for edoxaban than those of the other DOACs, which shows that higher the prescription rate of edoxaban, lower the rate of underdosing.

Since the appropriateness of DOAC dosing was evaluated based on various dose reduction criteria, such as US Food and Drug Administration labeling, the European Heart Rhythm Association practical guide, summaries of product characteristics, and manufacturer labeling recommendations, the prevalence of the inappropriate dosing of DOACs also varied depending on the research. However, the prevalence of underdosing tends to be higher in comparison with overdosing in all past reports, which is consistent with the results of the current study. It is likely that anticoagulant therapies are under-prescribed by physicians because of the fear of increased bleeding [3, 4, 21]. In addition, the prevalence of DOAC underdosing in Japan appears to be higher than in other countries. This may be because Asians have been reported to have a higher risk of intracranial hemorrhage during anticoagulant therapy with warfarin than non-Asians [22], thus physicians in Japan may have greater concerns regarding bleeding risk. Sato et al. revealed that the HAS-BLED score, which is a practical risk score for estimating the risk for major bleeding in AF patients, is an independent predictor of underdosing for apixaban (OR 1.59, 95% CI 1.18–2.13) and rivaroxaban (OR 2.27, 95% CI 1.51–3.39) [7]. This is in accordance with our results that the inappropriate dosing of DOACs was significantly associated with higher HAS-BLED scores (OR 1.87, 95% CI 1.42–2.51). In this study, a total of 20 cardiologists prescribed DOACs; however, the risk of stroke or hemorrhage in the patients varied for each physician (Additional file 1: Table S3), with the prevalence of inappropriate dosing also varying among physicians (Additional file 2: Figure S1). Guidelines for determining the recommended dose of DOACs taking into account real-world data are needed in the future.

Interestingly, our results demonstrated that the inappropriate dosing of DOACs was significantly higher in prescriptions for outpatients (vs. inpatients; OR 2.87, 95% CI 1.53–5.62, *p* < 0.001). For outpatients, there are two types of prescriptions in Japan: one is an out-of-hospital prescription for a community pharmacy and the other is an in-hospital prescription for the pharmacy inside the hospital. Recently, in Japan, more than 70% of the outpatients receive out-of-hospital prescriptions; this rate is over 95% at our hospital. For outpatients, the DOACs were all prescribed as out-of-hospital prescriptions during the study period. For inpatients, the physician’s prescription is audited by hospital pharmacists, and if the dose of the DOAC is considered inappropriate (i.e., underdosing or overdosing) in view of renal function, age, body weight, concomitant drug use, or other patient characteristics, a direct inquiry is performed by the hospital pharmacist. In contrast, for outpatients, there are few hospitals in Japan in which relevant clinical laboratory data are attached to out-of-hospital prescriptions, and community pharmacists generally cannot access these records. Since no laboratory data are displayed on the out-of-hospital prescriptions in our hospital, it is possible that adequate audits of community pharmacists for DOAC prescriptions have not been performed. This may be the reason why a greater proportion of inappropriate DOAC dosing was observed in outpatients compared with inpatients. In Japan, it has been reported that clinical laboratory data printed on out-of-hospital prescriptions contributes to an increase in the number of inquiries from community pharmacists to physicians in hospitals, thus promoting the optimization of prescriptions [23, 24]. For example, when community pharmacists noticed that the PT-INR of patients taking warfarin was rising, or levofloxacin was prescribed at a standard dose to patients with renal dysfunction, they may suggest that the corresponding physician reduce the dose appropriately. However, as prescriptions prior to inquiries conducted by the hospital and community pharmacists could not be analyzed owing to the retrospective nature of this study, it is unclear to what extent the pharmacist’s inquiries contribute to the appropriateness of the physician’s prescription. Chertow GM et al. demonstrated that a computerized order entry system considering renal function may improve dosing appropriateness and reduce the length of stay in inpatients with renal insufficiency [25]. In addition, it has been recommended that pharmacists check the laboratory data of outpatients in order to optimize prescriptions and play a more active role in aiding physicians to make better decisions [8]. In the future, further investigation of whether displaying the laboratory data on out-of-hospital prescriptions contributes to patient outcomes is warranted.

There are several limitations of this study that should be mentioned. First, this study was a retrospective, single-center pilot study, focused on prescriptions by only cardiologists in a Japanese university hospital. It is possible that prescriptions by non-cardiologists (e.g., brain surgeons) may have a lower rate of inappropriate DOAC dosing because they may also consider the risk of cerebral infarction rather than that of bleeding. Second, although we could extract clinical data at the time of the initial DOAC prescription, the number of patients who were continuously followed up at our hospital was not enough to investigate the association between the inappropriate dosing of DOACs and subsequent clinical outcome. As the association between the appropriateness of DOAC dosing and clinical outcomes among the four DOACs is still controversial, further studies are needed to clarify the effectiveness and safety of DOAC dosing considering real-world clinical data. Third, we could not investigate the factors associated with the inappropriate dosing of each DOAC despite differences in the dose reduction criteria of the four DOACs because of the relatively small number of patients. In the future, a multicenter study will be necessary to obtain an adequate sample size of patients to conduct similar studies in each DOAC group.

In conclusion, our results demonstrated that an inappropriate dose of a DOAC was prescribed in approximately 20% of AF patients, and occurred more frequently in outpatients (vs. inpatients) and in patients with a higher risk of bleeding. It is recommended that pharmacists play a greater role in assisting in the prescription process in order to help physicians make better decisions. In the future, it may be necessary to introduce a system that allows patient data, such as clinical laboratory data, to be reviewed at community pharmacies.

## Supplementary information

**Additional file1: Table S1.** Comparison of demographic characteristics among the 4 groups, underdosing, appropriate reduced dosing, overdosing, and appropriate standard dosing of direct oral anticoagulants. **Table S2**. Appropriateness of direct oral anticoagulants in previous published reports. **Table S3.** Distributions (%) of the risk scores for stroke or hemorrhage in patients by each physician who initiated direct oral anticoagulant therapy for more than 10 patients in the study period.

**Additional file 2: Figure S1.** Appropriateness of direct oral anticoagulant dosing for each cardiologist

## Data Availability

All data generated or analyzed during this study are included in this published article and its Supplementary information files.

## Abbreviations

AF: Atrial fibrillation
CrCl: Creatinine clearance
DOAC: Direct oral anticoagulant
GIH: Gastrointestinal hemorrhage
PT-INR: Prothrombin time-international normalized ratio
