# Supplementary material for: Inappropriate direct oral anticoagulant dosing in atrial fibrillation patients is associated with prescriptions for outpatients rather than inpatients: a single-center retrospective cohort study
Source: J Pharm Health Care Sci. 2020 Feb 11;6:2. doi: 10.1186/s40780-020-0157-z (PMC7014592; doi:10.1186/s40780-020-0157-z)
Supplement: Supplementary file 1 — Additional file1: Table S1. Comparison of demographic characteristics among the 4 groups, underdosing, appropriate reduced dosing, overdosing, and appropriate standard dosing of direct oral anticoagulants. Table S2. Appropriateness of direct oral anticoagulants in previous published reports. Table S3. Distributions (%) of the risk scores for stroke or hemorrhage in patients by each physician who initiated direct oral anticoagulant therapy for more than 10 patients in the study period. [file 40780_2020_157_MOESM1_ESM.docx]

Supplemental Table 1. Comparison of demographic characteristics among the 4 groups, underdosing, appropriate reduced dosing, overdosing, and appropriate standard dosing of direct oral anticoagulants.

| Characteristics | Underdosing  (n = 61) | Appropriate reduced dosing  (n = 116) | Overdosing  (n = 8) | Appropriate standard dosing  (n = 124) |
| --- | --- | --- | --- | --- |
| Age in year, median (IQR) | 67 (61–75) | 82 (76–86) | 79 (58–82) | 67 (61–75) |
| Male gender | 41 (67.2) | 50 (43.1) | 5 (62.5) | 96 (77.4) |
| Body weight in kg, median (IQR) | 61.6 (52.9–65.5) | 51.5 (44.1–56.8)^a^ | 58.7 (42.7–68.0) | 61.3 (52.2–65.5) |
| BMI in kg/m^2^, median (IQR) | 23.4 (21.5–24.9)^a^ | 21.6 (19.5–23.7)^a^ | 20.8 (18.2–25.5) | 24.0 (22.1–25.8) |
| Type of hospital visit |  |  |  |  |
| Inpatients | 15 (24.6) | 57 (49.1) | 4 (50.0) | 42 (33.9) |
| Outpatients | 46 (75.4) | 59 (50.9) | 4 (50.0) | 82 (66.1) |
| CrCl in mL/min, median (IQR) | 58.2 (50.5–71.4) | 42.3 (34.5–50.2)^a^ | 47.4 (41.8–77.3) | 76.8 (59.5–100.6) |
| Alcohol abuse | 3 (4.9) | 3 (2.6) | 0 (0) | 4 (3.2) |
| Smoking | 7 (11.5) | 11 (9.5) | 1 (12.5) | 21 (16.9) |
| History of warfarin use | 22 (36.1) | 35 (30.2) | 2 (25.0) | 31 (25.0) |
| History of bleeding | 11 (18.0) | 19 (16.4) | 3 (37.5) | 9 (7.3) |
| History of GIH | 4 (6.6) | 6 (5.2) | 1 (12.5) | 3 (2.4) |
| DOAC |  |  |  |  |
| Dabigatran | 6 (9.8) | 17 (14.7) | 0 (0) | 4 (3.2) |
| Rivaroxaban | 21 (34.4) | 21 (18.1) | 3 (37.5) | 59 (47.6) |
| Apixaban | 29 (47.5) | 40 (34.5) | 0 (0) | 44 (35.5) |
| Edoxaban | 5 (8.2) | 38 (32.8) | 5 (62.5) | 17 (13.7) |
| Comorbidities |  |  |  |  |
| Hypertension | 45 (73.8) | 82 (70.7) | 4 (50.0) | 75 (60.5) |
| Heart failure | 23 (37.7) | 41 (35.3) | 3 (37.5) | 32 (25.8) |
| Myocardial infarction | 11 (18.0) | 7 (6.0) | 1 (12.5) | 12 (9.7) |
| Dyslipidemia | 22 (36.1) | 36 (31.0) | 2 (25.0) | 43 (34.7) |
| Diabetes mellitus | 19 (31.2) | 25 (21.6) | 0 (0) | 33 (26.6) |
| Cerebrovascular disease | 13 (21.3) | 14 (12.1) | 1 (12.5) | 12 (9.7) |
| Hepatitis | 2 (3.3) | 4 (3.4) | 1 (12.5) | 8 (6.5) |
| Polypharmacy | 32 (52.5) | 55 (47.4) | 3 (37.5) | 36 (29.0) |
| Concomitant drug use |  |  |  |  |
| Antiplatelet drug | 21 (34.4) | 27 (23.3) | 1 (12.5) | 28 (22.6) |
| SAPT | 16 (26.2) | 21 (18.1) | 1 (12.5) | 16 (12.9) |
| DAPT | 3 (4.9) | 3 (2.6) | 0 (0) | 7 (5.7) |
| Non-SAPT/DAPT | 4 (6.6) | 4 (3.5) | 0 (0) | 7 (5.7) |
| NSAIDs | 0 (0) | 2 (1.7) | 1 (12.5) | 1 (0.8) |
| Amiodarone | 1 (1.6) | 5 (4.3) | 2 (25.0) | 3 (2.4) |
| Verapamil | 1 (1.6) | 0 (0) | 0 (0) | 2 (1.6) |
| Diltiazem | 1 (1.6) | 6 (5.2) | 0 (0) | 4 (3.2) |
| CHADS_2_, median (IQR) | 2 (1–3) | 2 (2–3) | 1 (1–3) | 1 (1–2) |
| 0–1 | 16 (26.2) | 28 (24.1) | 5 (62.5) | 70 (56.5) |
| ≥2 | 45 (73.8) | 88 (75.9) | 3 (37.5) | 54 (43.6) |
| CHA_2_DS_2_-VASc, median (IQR) | 4 (3–5) | 4 (3–5) | 3 (1–5) | 2 (1–4) |
| 0–1 | 1 (1.6) | 1 (0.9) | 2 (25.0) | 39 (31.5) |
| 2–3 | 24 (39.3) | 38 (32.8) | 3 (37.5) | 49 (39.5) |
| ≥4 | 36 (59.0) | 77 (66.4) | 3 (37.5) | 36 (29.0) |
| HAS-BLED, median (IQR) | 2 (1–3) | 2 (1–2) | 2 (1–3) | 1 (1–2) |
| 0–2 | 44 (72.1) | 91 (78.5) | 5 (62.5) | 107 (86.3) |
| ≥3 | 17 (27.9) | 25 (21.6) | 3 (37.5) | 17 (13.7) |

Missing data: ^a^ n-1.

Abbreviations: BMI, body mass index; CrCl, creatinine clearance; DAPT, dual antiplatelet therapy; GIH, gastrointestinal hemorrhage; IQR, interquartile range; NSAID, non-steroidal anti-inflammatory drug; SAPT, single antiplatelet therapy.

Supplemental Table 2. Appropriateness of direct oral anticoagulants in previous published reports.

| Study | Country | Study period | Number of  facilities | Criteria | Number of  patients | Standard dose | | Reduced dose | | Reference |
| --- | --- | --- | --- | --- | --- | --- | --- | --- | --- | --- |
|  |  |  |  |  |  | Appropriate | Inappropriate | Appropriate | Inappropriate |  |
| The Fushimi AF  Registry | Japan | March 2011 to  November 2015 | 80 | MLR | 539  (DAB/RIV/API) | 250 (46.4)  (Details unknown) | | 153 (28.4) | 136 (25.2) | 5 |
| The SAKURA AF  Registry | Japan | September 2013 to  December 2015 | 63 | MLR | 1658  (all 4 DOACs) | 746 (45.0) | 66 (4.0) | 477 (28.8) | 369 (22.2) | 6, 15 |
| Sato T, *et al*. | Japan | September 2011 to  January 2016 | 1 | MLR | 2272  (all 4 DOACs) | ND | 29 (1.3) | ND | 483 (21.3) | 7 |
| The ORBIT-AF II  Registry | US | February 2013 to  January 2016 | 242 | US FDA  labeling | 7925  (all 4 DOACs) | 6376 (80.5) | 260 (3.3) | 555 (7.0) | 734 (9.3) | 9 |
| The FANTASIIA  Registry | Spain | 1 June 2013 to  15 October 2014 | 50 | EHRA  guideline | 530  (DAB/RIV/API) | 217 (40.9) | 79 (14.9) | 141 (26.6) | 93 (17.5) | 16 |
| Lavoie K, *et al*. | Canada | October 2011 to  October 2014 | 1 | CSC  guideline | 500  (DAB/RIV/API) | 238 (47.6) | 51 (10.2) | 144 (28.8) | 67 (13.4) | 17 |
| McAlister FA, *et al*. | Canada | 2010 to 2015 | ND  (744 clinicians) | CSC  guideline | 6658  (DAB/RIV/API) | ND | 31 (0.5) | ND | 496 (7.4) | 18 |
| Moudallel S, *et al*. | Belgium | January 2016 to  December 2016 | 1 | SmPC | 755  (DAB/RIV/API) | ND | 49 (6.5) | ND | 131 (17.4) | 19 |
| Jacobs MS, *et al*. | Netherlands | 1 January 2012 to  13 December 2016 | 1 | ESC guideline  SmPC | 3231  (DAB/RIV/API) | ND | 147 (4.5) | ND | 174 (5.4) | 20 |

Abbreviations: API, apixaban; CSC, Canadian Society of Cardiology; DAB, dabigatran; EHRA, European Heart Rhythm Association; ESC, European Society of Cardiology, FDA, Food and Drug Administration; MLR, Manufacturer labeling recommendations; RIV, rivaroxaban; SmPC, summaries of product characteristics; US, united states.

ND: Not described.

Supplemental Table 3. Distributions (%) of the risk scores for stroke or hemorrhage in patients by each physician who initiated direct oral anticoagulant therapy for more than 10 patients in the study period.

|  |  | A | B | C | D | E | F | G | H | I | J | K | L |
| --- | --- | --- | --- | --- | --- | --- | --- | --- | --- | --- | --- | --- | --- |
|  |  | (n = 50) | (n = 38) | (n = 29) | (n = 26) | (n = 22) | (n = 21) | (n = 17) | (n = 14) | (n = 13) | (n = 12) | (n = 12) | (n = 11) |
| CHADS_2_ score | 0–1 | 32.0 | 52.6 | 41.4 | 34.6 | 40.9 | 28.6 | 38.9 | 21.4 | 53.9 | 46.7 | 33.3 | 54.6 |
|  | ≥2 | 68.0 | 47.4 | 58.6 | 65.4 | 59.1 | 71.4 | 61.1 | 78.6 | 46.2 | 53.3 | 66.7 | 45.5 |
| CHA_2_DS_2_-VASc score | 0–1 | 14.0 | 7.9 | 13.8 | 11.5 | 18.2 | 19.1 | 22.2 | 7.1 | 30.8 | 13.3 | 0 | 18.2 |
|  | 2–3 | 40.0 | 50.0 | 20.7 | 34.6 | 27.3 | 38.1 | 27.8 | 28.6 | 30.8 | 40.0 | 58.3 | 63.6 |
|  | ≥4 | 46.0 | 42.1 | 65.5 | 53.9 | 54.6 | 42.9 | 50.0 | 64.3 | 38.5 | 46.7 | 41.7 | 18.2 |
| HAS-BLED score | 0–2 | 80.0 | 76.3 | 86.2 | 69.2 | 59.1 | 90.5 | 72.2 | 85.7 | 92.3 | 86.7 | 83.3 | 90.9 |
|  | ≥3 | 20.0 | 23.7 | 13.8 | 30.8 | 40.9 | 9.5 | 27.8 | 14.3 | 7.7 | 13.3 | 16.7 | 9.1 |
