# Supplementary material for: Inappropriate direct oral anticoagulant dosing in atrial fibrillation patients is associated with prescriptions for outpatients rather than inpatients: a single-center retrospective cohort study
Source: J Pharm Health Care Sci. 2020 Feb 11;6:2. doi: 10.1186/s40780-020-0157-z (PMC7014592; doi:10.1186/s40780-020-0157-z)
Supplement: Supplementary file 2 — Additional file 2: Figure S1. Appropriateness of direct oral anticoagulant dosing for each cardiologist [file 40780_2020_157_MOESM2_ESM.pptx]

## Slide 1
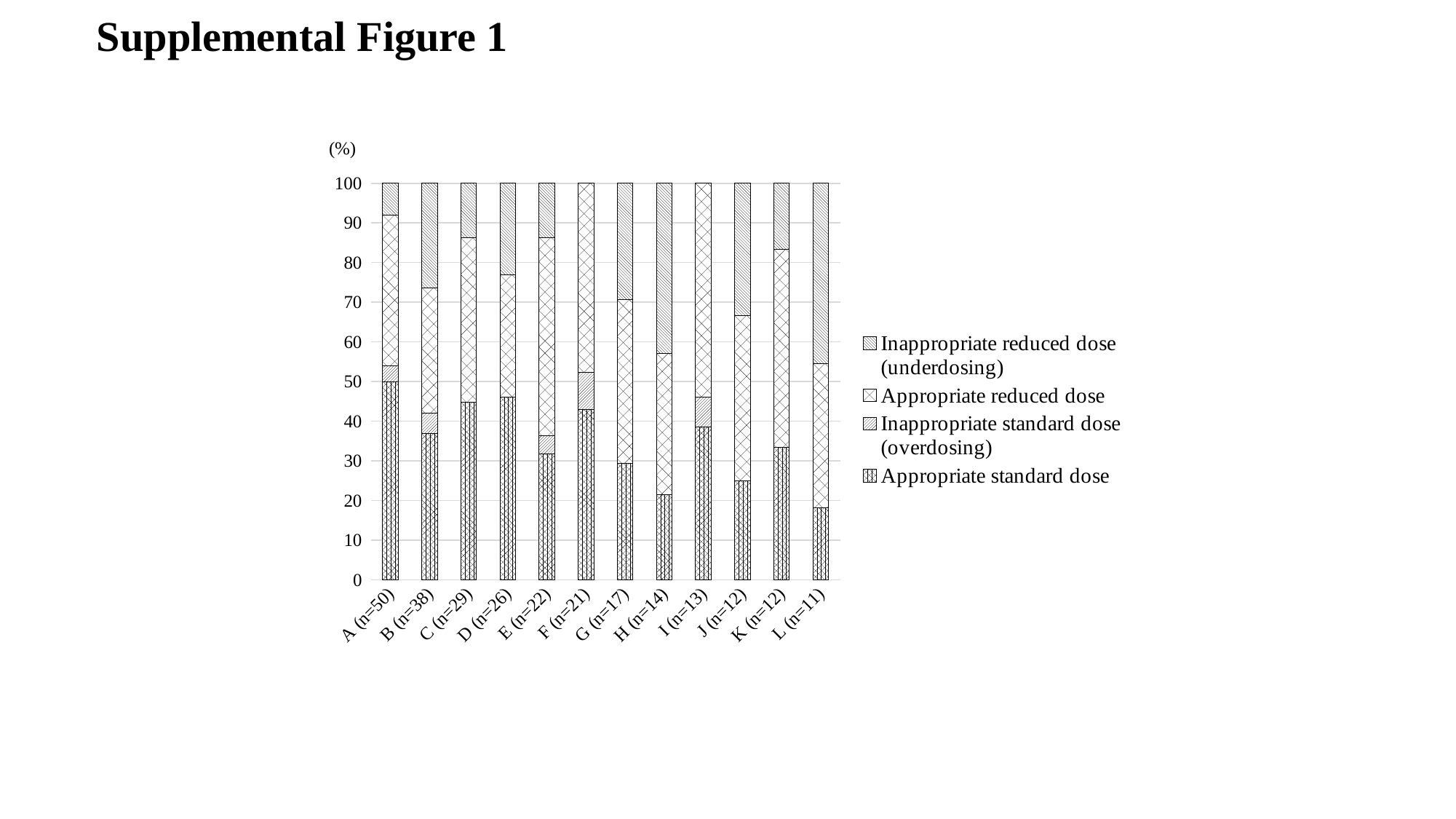

Supplemental Figure 1
(%)
### Chart
| Category | Appropriate standard dose | Inappropriate standard dose (overdosing) | Appropriate reduced dose | Inappropriate reduced dose (underdosing) |
|---|---|---|---|---|
| A (n=50) | 50.0 | 4.0 | 38.0 | 8.0 |
| B (n=38) | 36.84210526315789 | 5.263157894736842 | 31.57894736842105 | 26.31578947368421 |
| C (n=29) | 44.827586206896555 | 0.0 | 41.37931034482759 | 13.793103448275861 |
| D (n=26) | 46.15384615384615 | 0.0 | 30.76923076923077 | 23.076923076923077 |
| E (n=22) | 31.818181818181817 | 4.545454545454546 | 50.0 | 13.636363636363635 |
| F (n=21) | 42.857142857142854 | 9.523809523809524 | 47.61904761904761 | 0.0 |
| G (n=17) | 29.411764705882355 | 0.0 | 41.17647058823529 | 29.411764705882355 |
| H (n=14) | 21.428571428571427 | 0.0 | 35.714285714285715 | 42.857142857142854 |
| I (n=13) | 38.46153846153847 | 7.6923076923076925 | 53.84615384615385 | 0.0 |
| J (n=12) | 25.0 | 0.0 | 41.66666666666667 | 33.33333333333333 |
| K (n=12) | 33.33333333333333 | 0.0 | 50.0 | 16.666666666666664 |
| L (n=11) | 18.181818181818183 | 0.0 | 36.36363636363637 | 45.45454545454545 |
